# Exploration of Human Salivary Microbiomes—Insights into the Novel Characteristics of Microbial Community Structure in Caries and Caries-Free Subjects

**DOI:** 10.1371/journal.pone.0147039

**Published:** 2016-01-19

**Authors:** Jianye Zhou, Nan Jiang, Shaoguo Wang, Xiaopan Hu, Kangli Jiao, Xiangyi He, Zhiqiang Li, Jizeng Wang

**Affiliations:** 1 College of Civil Engineering and Mechanics, Lanzhou University, Lanzhou, 730000, Gansu, China; 2 State Key Laboratory of Forest and Soil Ecology, Institute of Applied Ecology, Chinese Academy of Sciences, Shenyang 110016, Liaoning, China; 3 School of Stomatology, Lanzhou University, Lanzhou 730000, Gansu, China; 4 Key Laboratory of Oral Diseases of Gansu Province, School of Stomatology, Northwest University for Nationalities, Lanzhou 730030, Gansu, China; Universidad Miguel Hernandez, SPAIN

## Abstract

Recently, high-throughput sequencing has improved the understanding of the microbiological etiology of caries, but the characteristics of the microbial community structure in the human oral cavity with and without caries are not completely clear. To better understand these characteristics, Illumina MiSeq high-throughput sequencing was utilized to analyze 20 salivary samples (10 caries-free and 10 caries) from subjects from the same town in Dongxiang, Gansu, China. A total of 5,113 OTUs (Operational Taxonomic Units, 97% cutoff) were characterized in all of the salivary samples obtained from the 20 subjects. A comparison of the two groups revealed that (i) the predominant phyla were constant between the two groups; (ii) the relative abundance of the genera *Veillonella*, *Bifidobacterium*, *Selenomonas*, *Olsenella*, *Parascardovia*, *Scardovia*, *Chryseobacterium*, *Terrimonas*, *Burkholderia* and *Sporobacter* was significantly higher in the group with caries (P < 0.05); and (iii) four genera with low relative abundance (< 0.01% on average), including two characteristic genera in caries (*Chryseobacterium* and *Scardovia*), significantly influenced the microbial community structure at the genus and OTU levels. Moreover, via co-occurrence and principal component analyses, the co-prevalence of the pathogenic genera was detected in the caries samples, but in the caries-free samples, the function of clustered genera was more random. This result suggests that a synergistic effect may be influencing the assembly of the caries microbial community, whereas competition may play a more dominant role in governing the microbial community in the caries-free group. Our findings regarding the characteristics of the microbial communities of the groups with and without caries might improve the understanding of the microbiological etiology of caries and might improve the prevention and cure of caries in the future.

## Introduction

Dental caries, which is mainly caused by bacteria, is a chronic progressive infectious disease in humans and is characterized by high occurrence and wide distribution [1]. Caries not only leads to stomatological system dysfunction but also to a variety of other systemic disease risk factors, such as digestive disorders, cardiovascular disease and kidney damage [2, 3]. "In the next 10 years, we should focus on strengthening the research of the impact” of caries, which is the most widely distributed disease and seriously affects the quality of life of those it affects [4]. Thus, the prevention of dental caries is a very important task. Without effective prevention and treatment, caries will continue to seriously affect human health.

With the development of high-throughput sequencing technologies, the results of related studies in recent years have suggested that the composition of the bacterial community may be much more important to the development of caries than the presence of a single potentially pathogenic species [5]. Caries was reported to have a more varied microbial community structure [6, 7]. Nevertheless, the investigations on the relationship between the microbial community structure and the incidence of dental caries using high-throughput sequencing technologies were more focused on finding differences in the microbial genera, such as *Lactobacillus*, *Rothia*, *Granulicatella*, *Actinomyces*, *Gemella*, *Haemophilus*, and *Veillonella* [6–10], between caries and caries-free subjects. However, the results did not sufficiently explain the characteristics of the microbial community structure. Moreover, the various genera and species in the results reported were not always identical. Therefore, the microbial community structure of caries was more complicated than what we had previously described [11] and thus required a more in-depth exploration.

As has been reported, the living environments of people, including diet, lifestyle, water, soil and other environmental factors, affect the oral microbial community structure and the quantity of flora and microbial virulence [12, 13]. Consequently, to eliminate the influence of these factors in this study, 20 volunteers (10 with caries and 10 without caries) with the same living environment and habits were selected from the same town in Dongxiang, Gansu, China. The salivary bacterial communities of the subjects were investigated using the Illumina MiSeq platform. The similarities and differences in the bacterial community diversity, composition and co-prevalence between samples with and without caries were analyzed, providing new insights into microbiological etiology and a new perspective of the prevention of caries.

## Materials and Methods

### Subjects and specimen collection

All human host volunteers were recruited from the town of Nalesi in the Dongxiang autonomous county of Gansu Province in China in September 2013. The oral health status of all individuals was determined by a dentist who performed a full-mouth clinical examination that included inspection of the teeth, oral mucosa and periodontal tissues as well as a review of medical and dental histories [14]. The subjects excluded from this study were those (i) whose age did not fall between 28–55 years old; (ii) who had immune suppression or systemic diseases (such as diabetes and HIV); (iii) who had received systemic periodontal treatment in the preceding year or had taken antibiotics within the previous 3 months; and/or (iv) who were pregnant or smokers. Finally, 10 caries subjects (C) and 10 caries-free subjects (H) were selected (Table 1). The subjects were fully informed about the research objectives and signed an informed consent form with the approval of the Ethics Committee of Northwest University for Nationalities.

**Table 1:** Characteristics of subjects and DMFT index in this study.

Subject ^#^	Gender	Age	DMFT *
H-1	F	37	0
H-2	M	28	0
H-3	F	36	0
H-4	M	29	0
H-5	M	34	0
H-6	M	41	0
H-7	M	40	0
H-8	F	31	0
H-9	F	43	0
H-10	M	41	0
C-1	F	44	10
C-2	F	34	4
C-3	M	25	4
C-4	M	38	6
C-5	M	37	5
C-6	M	33	4
C-7	F	36	6
C-8	M	36	5
C-9	F	55	9
C-10	F	53	10

* DMFT: Decayed, Missing and Filled Teeth.

^#^ H and C represent caries-free and caries subjects, respectively.

### DNA extraction, library construction and sequencing

Salivary DNA was extracted using the Qiagen Stool Mini Kit (Qiagen, Valencia, CA) [14], according to the method described [15], with slight modification. Each 1 mL unstimulated salivary sample was collected using an asepsis tube containing 500 μL of TE buffer (25 mM Tris HCl, 10 mM EDTA, pH 8.0) from a person who had not brushed his/her teeth, gargled or eaten within the past 12 h. The samples were taken to the lab immediately after the clinical parameters were recorded.

To determine the diversity and composition of the bacterial community in each salivary sample, PCR amplifications of the bacterial 16S rRNA V4 region (approximately 240 bp) were performed using the PCR-based protocol described and the forward and reverse primers (5'-AYTGGGYDTAAAGNG-3' and 5'-TACNVGGGTATCTAATCC-3', respectively) [16]. To allow simultaneous analyses of several samples in a single sequencing run, each sample was tagged by adding an index sequence extension at the 5' end of the forward primers for multiplexing. The PCR amplification was performed in triplicate using an Eppendorf Mastercycler ep gradient thermal cycler (Eppendorf, Hauppauge, New York) in a total volume of 25 μL containing 9 μL of sterilized water, 5 μL of 5× PCR buffer, 5 μL of 5× PCR GC high enhancer, 2 μL of dNTP (2.5 mM), 2 μL of template DNA (200 ng/μL), 0.25 μL of TaKaRa polymerase (5 U/μL) and 1 μL of each primer (10 μM). The thermal cycling conditions were as follows: an initial denaturation at 98°C for 5 min, followed by 27 cycles at 98°C for 30 s, 50°C for 30 s, and 72°C for 30 s, with a final extension at 72°C for 5 min. Following the amplification, 3 μL of PCR product was used for agarose gel (2.0%) detection. The triplicate PCR reactions for each sample preparation were combined and quantified with PicoGreen using a FLUOstar Optima (BMG Labtech, Jena, Germany). The PCR product from each sample was pooled with those of the other samples for one sequencing run. After quality control using Agilent 2100, the pooled mixture was re-quantified with PicoGreen. The purified mixture was diluted and denatured to obtain a 5 pM sample DNA library, which was then loaded on a 500-cycle (2x250 paired ends) kit and run on the Illumina MiSeq platform.

All raw sequences were trimmed using the Quantitative Insights Into Microbial Ecology (QIIME) toolkit v.1.5.0 [16]. Next, the reads that were shorter than 200 bp, contained any ambiguous bases, or exhibited a homopolymer longer than 10 bp were removed. The operational taxonomic units (OTUs) were clustered with a 97% similarity cutoff using UPARSE (version 7.1 http://drive5.com/uparse/), and the chimeric sequences were identified and removed using UCHIME [17]. Representative sequences for each OTU were assigned taxonomically using the Ribosomal Database Project (RDP) classifier [18] and the BLAST tool of the Human Oral Microbiome Database (HOMD; http://www.homd.org/) [5].

### Statistical analysis

Sampling effectiveness was evaluated by rarefaction curves that were drawn from a raw OTU table. The Shannon‒Wiener, Simpson [19], Chao and ACE [20] indices were calculated for the measurement of α-diversity. Good’s coverage [21] was calculated as G = 1−n/N, where n is the number of singleton phylotypes and N is the total number of sequences in the sample. To estimate β-diversity, the Bray-Curtis (abundance-based) and weighted UniFrac (phylogenetic-based) distance matrices were calculated. An estimation of all α-diversity indices, rarefaction curves and the weighted UniFrac distance matrix was performed using QIIME. Comparison of β-diversity between H and C was performed by Constrained Analysis of Principal Coordinates (CAP), Analysis of Similarities (ANOSIM) [22] and the Multiple Response Permutation Procedure (MRPP) [23, 24] using the R package vegan [25]. We tested for nonrandom patterns of taxon co-occurrence by calculating the C-scores [26] for the presence/absence matrix of all genera, which was performed on the program EcoSim (http://www.uvm.edu/~ngotelli/EcoSim/EcoSim.html). This measurement is compared with a null distribution of the random matrices of the same size. If the observed C-score is significantly larger than the score for the null hypothesis, it suggests segregation between taxa, and if the observed C-score is significantly smaller than the score for the null hypothesis, it suggests aggregation between taxa. Schoener's index (abundance-based; measuring the co-occurrence probability of two taxa) [27], which was computed for each pair of genera using the R spaa package [28], was used to draw a heatmap for evaluating the distribution pattern at the genus level in the two groups using the ggplots package in R [29]. A Principal Components Analysis (PCA) was also performed using the R package vegan to discover the relationship between the genera in the two groups. Redundancy analysis (RDA) was performed using the vegan package in R to test the effects of low-abundance genera on the microbial community structure at the genus and OTU levels.

LEfSe (Linear discriminant analysis Effect Size) was used to determine the taxa characterizing the differences in the bacterial composition between the samples with and without caries [30–32]. Specifically, the non-parametric factorial Kruskal-Wallis sum-rank test was used to detect taxa with significant differential abundance; a set of pairwise tests among subclasses, if any existed, was performed using the Wilcoxon rank-sum test; and Linear Discriminant Analysis was used to estimate the effect size of each differentially abundant taxa [32].

Sequence data have been submitted to the GenBank databases under the biosample accession Nos. SAMN03073526—SAMN3073545.

## Results and Discussion

### Bacterial diversity and structure of the salivary samples of caries and caries-free subjects

After parallel sequencing of the 20 salivary samples and sequence quality control, we obtained a total of 252,757 high-quality reads with a mean read length of 220 bp. Clustering of all of the qualified sequences at the 97% similarity level generated a total of 5,113 OTUs. From the healthy and caries samples, 2684 and 3703 OTUs were detected, respectively, and 24.9% of the total OTUs were shared by the two groups. The average level of Good’s coverage per sample was over 97%, suggesting that the 16S rRNA gene sequences identified represent the majority of bacterial taxa inhabiting the saliva in the current study.

Four diversity indices were calculated from each salivary sample, and an α-diversity comparison indicated that caries and caries-free subjects were did not significantly differ between healthy and caries subjects (S1 Table). These results agree with previous findings in a plaque bacterial microbiome diversity study in children with or without caries [33] and with Yang’s results via pyrosequencing analysis on the saliva of adults between 18 and 22 years old [7], in which the caries and caries-free groups exhibited a similar level of diversity. However, some previous findings in adults by HOMIM analysis on stimulated whole saliva [34] and in children by pyrosequencing analysis on supragingival plaques [35] showed that the caries reduced the bacterial community diversity [35]. It was proposed that the richness may have been lower with lower oral pH [36], a condition that is more prone to dental caries. It has also been reported that “initiation of oral infectious diseases and disease status are associated with increased diversity and richness of the microbiota” [37]. Therefore, with different samples and methods, the previous results of the bacterial community diversity of caries and caries-free subjects would be changed, which requires further study on larger samples and on microenvironmental factors such as pH.

**Fig 1:**
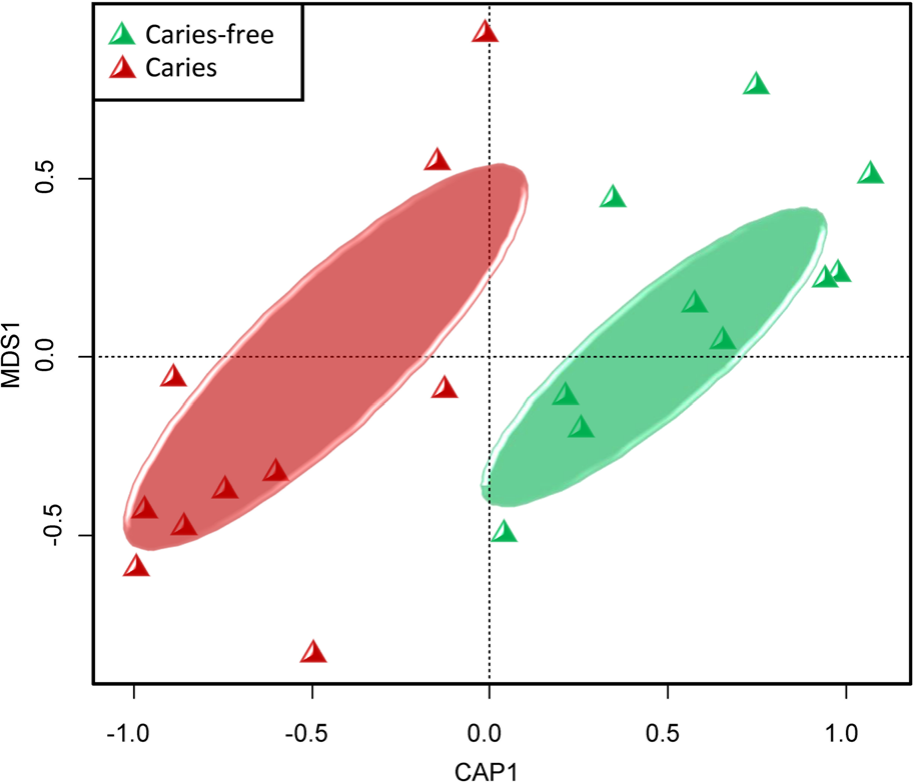
Constrained Analysis of Principal Coordinates (CAP) plots (based on weighted UniFrac) estimating the β-diversity variation among the caries and caries-free groups.

When comparing the bacterial community structure, we first observed that caries and caries-free samples separated well in CAP representations of the phylogenetic metric (Fig 1). Then, ANOSIM and MRPP were performed, and the results showed that the bacterial composition in the two groups was phylogenetically distinct (ANOSIM: R = 0.145, MRPP: A = 0.033, P<0.05), providing robustness of the ordination patterns displayed in the CAP plot. The bacterial community composition of the caries and caries-free samples was notably different, and the variations in bacterial community composition may cause caries [7, 11]. Using pair-wise UniFrac distances, caries microbiomes were reported to be more variable in community structure, whereas the healthy microbiomes were more similar to each other [6, 7]. However, no significant difference was detected between healthy and caries individuals in our study (P > 0.05). One explanation is the sampling location. Microbiomes in the oral cavity are dependent on the local microecological conditions [38]. In this study, saliva rather than supragingival plaque was collected. Different atmospheric conditions culture different bacterial community structure [38]. Saliva is an easily acquired, relatively stable, inexpensively accessible biological fluid that has been thoroughly analyzed for biomarkers of health and disease for a long period [34, 39, 40]. However, it should be noted that saliva may not be representative of the bacterial diversity located at the disease site [41], which must be addressed in further studies and clinical applications regarding caries. In addition, the stages of caries progression must be considered. In this study, DMFT was at least 4, and in another study using salivary samples, caries bacterial community structure was more variable when DMFT was not less than 6 [7]. Therefore, other stages of caries should receive more attention in the future.

### Classification of taxa among the salivary samples of caries and caries-free subjects

The taxonomic assignments of all OTUs resulted in 27 phyla, with a vast majority of OTUs belonging to eight major phyla (>0.5% on average; >95% overall): *Proteobacteria* (relative abundance: 12.1‒53.7%), *Firmicutes* (19.0‒51.1%), *Actinobacteria* (6.9‒23.5%), *Bacteroidetes* (0.8‒19.9%), *Fusobacteria* (0.03‒16.9%), *Spirochaetes* (0.2‒8.1%), *TM7* (0.06–2.9%) and *Cyanobacteria* (0.02–2.4%). The occurrences of the most common phyla in both groups are the same, a finding that is largely comparable to that found in previous studies [42–44]. The phyla proportions may differ in these studies; however, the most common phyla in oral saliva are relatively constant [7, 14]. *Cyanobacteria* are ubiquitous in water [45], and several papers have reported the presence of these bacteria in the oral cavity [7, 42]. We previously proposed that these bacteria may be ingested through drinking water or by consuming contaminated seafood [45]. This presumption has been supported by Benitez-Paez et al. [46], where olive oil chloroplasts were amplified with 16S universal primers. Therefore, it is relevant to consider potential activities caused by environmental microorganisms. The associative distinctions may well be minimized by our sampling approach: 20 volunteers selected from the same town with the same living environment and habits.

The sequences were further divided into 60 classes, 95 orders, 157 families and 303 genera. The compositions of major bacteria from the class to genus taxonomic levels of the saliva samples of caries and caries-free subjects are shown in S1 Fig. It should be noted that some bacterial genera detected such as *Thermus* and *Caulobacter* are environmental rather than oral. In addition to acquisition from the external environment, another explanation is that these hits searched against the RDA 16S rRNA gene database may correspond to related genera without being those clearly environmental bacteria.

### Identification of the characteristic bacterial taxa of caries and caries-free subjects

**Fig 2:**
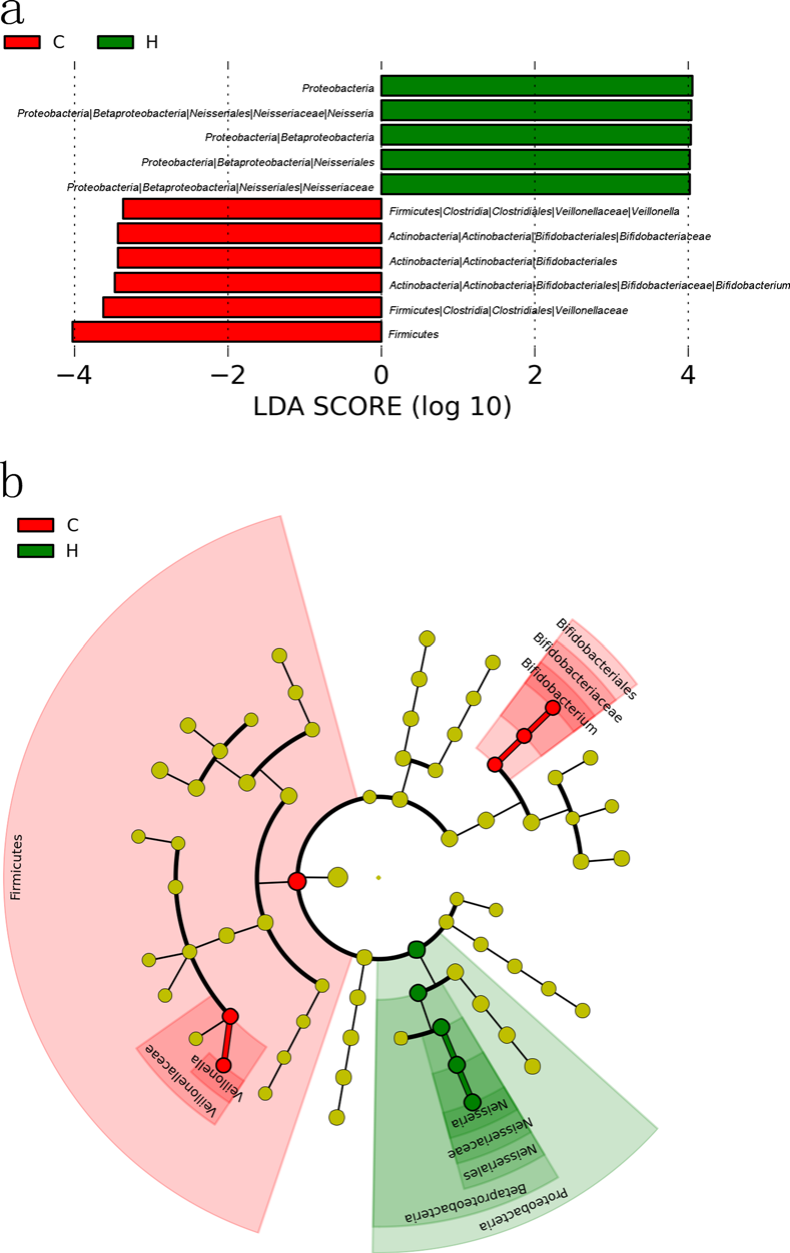
The dominant taxa in the caries (C) and caries-free (H) groups were analyzed using LEfSe (P<0.05). The average relative abundance of each clade was more than 0.05%. (a) Visualization of differential taxa ranked by effect size. (b) Representation of relevant taxa on a phylogenetic tree.

The differences in the relative abundance between caries and caries-free subjects could be found at several taxonomic levels (S1 Fig). Thus, the LEfSe method was performed to graphically represent the characteristic bacteria with their effect sizes or in a taxonomic tree. This method detected 11 differentially abundant taxonomic clades with LDA scores higher than 2.0 (P<0.05). The relative abundance of *Firmicutes*, especially the genus *Veillonella* (belonging to class *Clostridia*, order *Clostridiales*, family *Veillonellaceae*) was higher in samples with caries (Fig 2). A higher value in samples with caries was also detected within the genus *Bifidobacterium*, the family *Bifidobacteriaceae* and the order *Bifidobacteriales*, all of which belong to the phylum *Actinobacteria* (Fig 2). As Fig 2 shows, the average relative abundance of *Proteobacteria*, especially the genus *Neisseria* (belonging to class *Betaproteobacteria*, order *Neisseriales*, family *Neisseriaceae*), was higher in samples from the caries-free subjects. As in a previous study [7], the suspected cariogenic bacteria, such as *Streptococcus* or *Lactobacillus* harboring acidogenic potential, were again absent from the differences. One hypothesis is that acid-producing bacteria are the vehicle for penetrating the enamel and only appear in deep dentin caries [47]. *Veillonella* are obligate anaerobic Gram-negative bacteria and were originally isolated from the oral cavity and intestinal tract of humans and animals [48]; these bacteria may serve as an “acid sink”, allowing acidogenic bacteria such as *Streptococcus* species to grow and continue to produce additional acid [49, 50]. Thus, our data support the notion that the genus *Veillonella* is related to cariogenic potential [51, 52]. *Bifidobacterium* is a Gram-positive genus. Some bifidobacteria are used as probiotics [53], and some have been reported to show high relative abundance in caries subjects [6]. Moreover, this genus has been found to be associated with different stages of caries [6, 54–56]. The results suggested that we should further develop related culture and biochemical research to identify the species, not just by sequencing, to confirm the relationship between this genus and caries.

In addition, using the LEfSe method (P<0.05), the relative abundance of another 8 “minor genera” (average relative abundance < 0.05%), including *Selenomonas*, *Olsenella*, *Parascardovia*, *Scardovia*, *Chryseobacterium*, *Terrimonas*, *Burkholderia* and *Sporobacter*, was also significantly higher in the saliva samples of caries subjects than in those of caries-free samples (S2 Fig). These minor genera were also supposed as characteristic genera in caries. There has been no complete consensus obtained from any two papers concerning the microbiomes in health and caries [6, 7, 47, 49, 54, 55, 57], consistent with the “Ecological Plaque Hypothesis” that suggested caries as a consequence of imbalances in the resident microflora [58].

Last but not least, we investigated the influence of all the low-abundance genera (< 0.01%) on the microbial community structure in oral cavity by RDA, in which all the low-abundance genera were regarded as separate impact factors. Four genera, including *Bacillus*, *Sneathia*, *Chryseobacterium*, and *Scardovia*, influenced the microbial community structure at the genus and OTU levels (Table 2). It also should be noted that two characteristic genera in caries (*Chryseobacterium* and *Scardovia*) were included. Given good reproducibility and significance, metagenomic analysis of 16S rRNA identified species belonging to *Chryseobacterium* and *Scardovia* as important. It has been reported that in animal models, certain low-abundance microbial pathogens can orchestrate disease by remodeling a normally benign microbiota into a dysbiotic one. All of these results support the idea that some bacteria in the oral cavity may disrupt the host microbial homeostasis to cause caries, similar to a previous report in inflammatory disease periodontitis [59]. Further experimental analysis in animal models should be carried out to validate this hypothesis.

**Table 2:** Descriptive scale illustrating the effects of genera with low relative abundance (< 0.01%) on the bacterial community structure at the genus and OTU levels.

	Genus level		OTU level	
Taxa	r^2^	P	r^2^	P
*Firmicutes_Bacilli_Bacillales_Bacillaceae_Bacillus*	0.9863	0.042*	0.9892	0.046*
*Fusobacteria_Fusobacteria_Fusobacteriales_Leptotrichiaceae_Sneathia*	0.9863	0.042*	0.9892	0.046*
*Bacteroidetes_Flavobacteria_Flavobacteriales_Flavobacteriaceae_Chryseobacterium*	0.4209	0.006**	0.3481	0.015*
*Actinobacteria_Actinobacteria_Bifidobacteriales_Bifidobacteriaceae_Scardovia*	0.7388	0.002**	0.7660	0.001***

Significance codes:

‘***’ 0.001

‘**’ 0.01

‘*’ 0.05 P values based on 999 permutations.

### Co-occurrence analysis reveals community structures in samples from caries and caries-free subjects

To further explore the community structure, a co-occurrence analysis was performed for each group at the genus level. First, we used the C-score of co-occurrence analysis, which compares the taxon distribution of a dataset (using the presence/absence of the datasets that were transformed from the abundance data) to a randomized distribution of the same number of taxa. The observed C-score was significantly different from the null hypothesis (random distribution) when the genera of caries-free subjects were analyzed (observed C = 1.367, expect C = 1.332; P<0.005). In addition, when the genera from the samples with caries were analyzed, the C-score indicated that the communities also displayed co-occurrence patterns significantly different from the null hypothesis (observed C = 1.403, expect C = 1.381; P<0.05). These results suggested that there was a synergistic effect (or possible competition) of genera in both groups.

**Fig 3:**
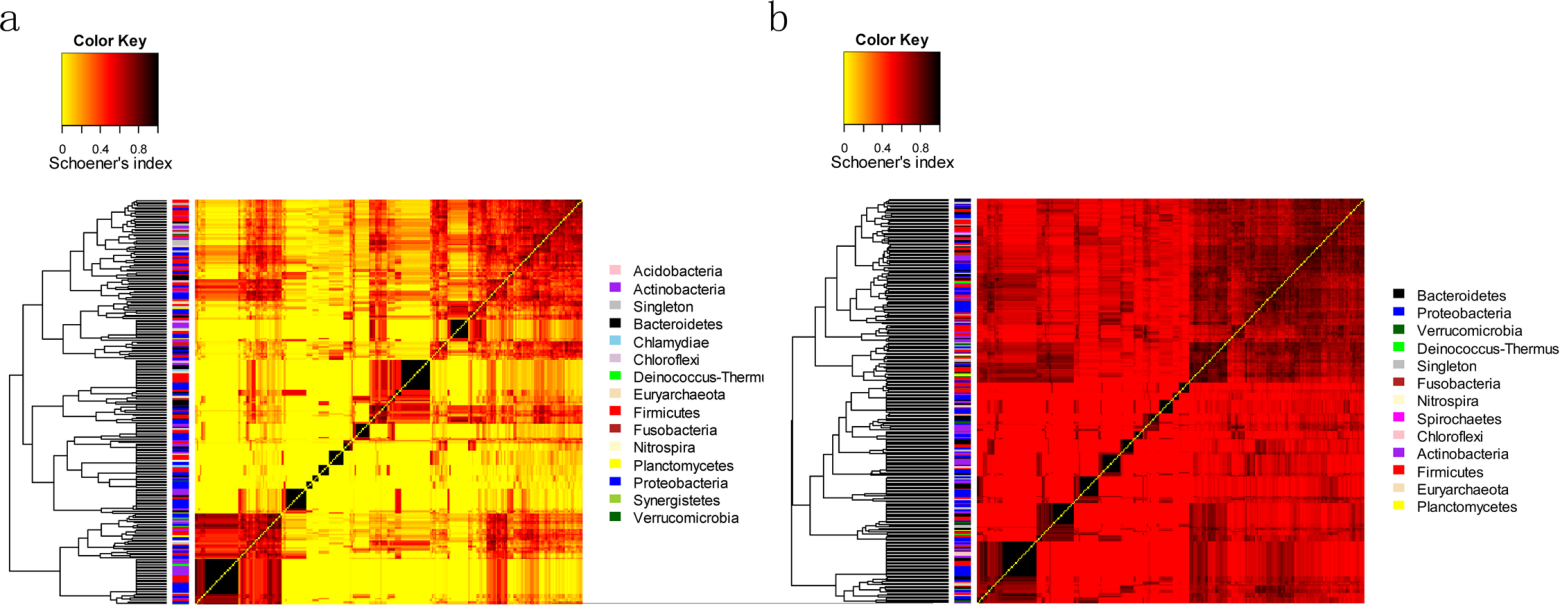
Heatmaps displaying genus distribution patterns within caries-free (a) and caries (b) groups. The clustering and heatmaps were computed from Schoener’s index matrix. The Schoener’s index matrices calculated from each group are found in S2 and S3 Tables.

Schoener's abundance-based co-occurrence index was further calculated for each pair of genera (detailed information available as S2 and S3 Tables), and we drew heatmaps from the resultant Schoener's index matrices (S2 and S3 Tables) to separately assess the distribution pattern of genera within each group. In each group, all genera were disproportionally grouped into two clusters from the root of the dendrogram based on the similarity of their co-occurrence probabilities (Fig 3). However, the relatively high co-occurrence probabilities (Schoener's indices) were more frequently observed in the genera belonging to *Proteobacteria*, *Firmicutes* and *Actinobacteria* with respect to the caries-free subjects (Fig 3A). By contrast, the genera from the subjects with caries that were prone to high co-occurrence probabilities were spread throughout more phyla (Fig 3B). A significantly higher value of Schoener’s index was detected in the subjects with caries (S3 Fig). These scenarios suggested that a particular different distribution pattern (synergistic effect or competition) may be characteristic of the genera from caries and caries-free subjects.

**Fig 4:**
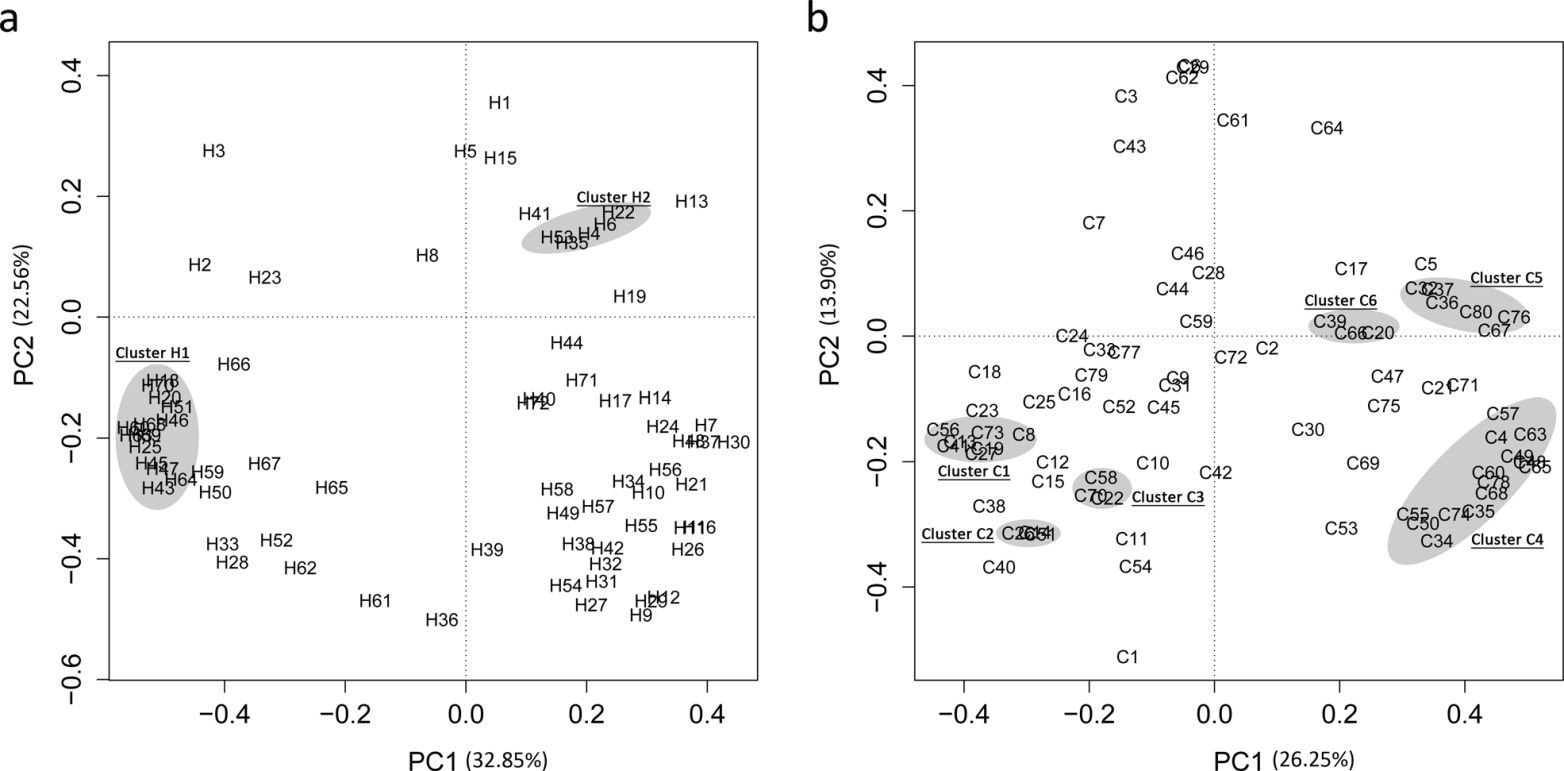
Principal Component Analysis (PCA) of the distribution pattern at the genus level of caries-free (a) and caries (b) groups, respectively. The shadows represent the distance between the genera. The taxa corresponding to the numbers are listed in S5 Table.

To investigate the distribution patterns at the genus level (average relative abundance > 0.01%), PCA was then performed in each group (Fig 4 and S5 Table), and the details are provided in S4 Table. Interestingly, the co-prevalence of pathogenic genera was always detected in the caries group (Fig 4B), including the pathogens reported previously (such as *Actinobacillus* (C4), *Micrococcus* (C74), and *Acinetobacter* (C34)) and potential pathogens in the present study (such as *Bifidobacterium* (C13), *Veillonella* (C8), and *Selenomonas* (C19)). Schoener’s index was also higher (all more than 0.75) between these pathogenic genera in the caries group (Table 3). However, the function of the clustered genera was more random in the caries-free group than in the caries group (Fig 4A and S4 Table). For example, the shadow in Fig 4A included probiotics (such as *Parascardovia* (H90) and *Weissella* (H20)), potential pathogens (such as *Herbaspirillum* (H51) and *Micrococcus* (H64)) and some genera with unknown function (such as *Thermus* (H25) and *Caulobacter* (H47)). The “co-prevalence” phenomenon of a genus or species had been noticed recently in caries but was usually based on the content or numbers of the genus [52, 54, 60]. In this study, we enhanced the analyses based on the co-occurrence index (Schoener’s index) and reduced the number of dimensions (PCA). Our results were consistent with the previous report that a given bacterial consortium may be associated with either cariogenic or non-cariogenic conditions [61]. The ecological plaque hypothesis proposes that the disease is the result of a shift in the ecological balance of dental plaque towards a more cariogenic flora [58]. The results of our study further indicated that a synergistic effect may be predominant in the caries microbial community structure but that competition played a dominant role in the caries-free microbial community structure. In other words, the competition in the microbial community structure of the oral cavity is the dominant factor for “balance” in the community; when the competition converts to a synergistic effect, the “balance” of the microbial community structure of the oral cavity is broken, which can lead to disease, such as caries [62]. However, because the present study involved sequencing, whether and how the distribution pattern (synergistic effect or competition) functions in the cariogenic potential requires further study, such as experimentation on animals.

**Table 3:** Schoener index values between the different genera from the caries-free (upper triangular matrix) or the caries (lower triangular matrix) samples.

	*Actinobacillus*	*Micrococcus*	*Acinetobacter*
*Actinobacillus*	-	0.36	0.61
*Micrococcus*	0.83	-	0.53
*Acinetobacter*	0.86	0.87	-
	*Bifidobacterium*	*Veillonella*	*Selenomonas*
*Bifidobacterium*	-	0.63	0.67
*Veillonella*	0.79	-	0.65
*Selenomonas*	0.86	0.84	-

## Supporting Information

S1 Fig Taxonomic resolutions for the classifiable bacterial sequences at the class level (a), order level (b), family level (c) and genus level (d) of caries (C) and caries-free (H) samples. (TIF)

S2 Fig The characteristic bacterial genera, including *Selenomonas* (a), *Olsenella* (b), *Parascardovia* (c), *Scardovia* (d), *Chryseobacterium* (e), *Terrimonas* (f), *Burkholderia* (g), and *Sporobacter* (h), were analyzed using LEfSe across the caries (C) and caries‑free (H) samples. Solid lines and dotted lines represent the means and medians, respectively.
(TIF)

S3 Fig Co-occurrence probabilities (based on Schoener’s index) of caries-free (H) and caries (C) samples at the genus level (P<0.001). (TIF)

S1 Table Four diversity indices were calculated from each salivary sample. (XLSX)

S2 Table Schoener's indices calculated for all pairs of genera found in the caries-free group. (XLSX)

S3 Table Schoener's indices calculated for all pairs of genera found in the caries group. (XLSX)

S4 Table The details of the genera clustered by PCA (Fig 4). (XLSX)

S5 Table The taxa corresponding to the numbers in Fig 4. (XLSX)

S6 Table References cited in S4 Table. (XLSX)
