# Supplementary figures and images for: Exploration of Human Salivary Microbiomes—Insights into the Novel Characteristics of Microbial Community Structure in Caries and Caries-Free Subjects
Source: PLoS One. 2016 Jan 19;11(1):e0147039. doi: 10.1371/journal.pone.0147039 (PMC4718657; doi:10.1371/journal.pone.0147039)

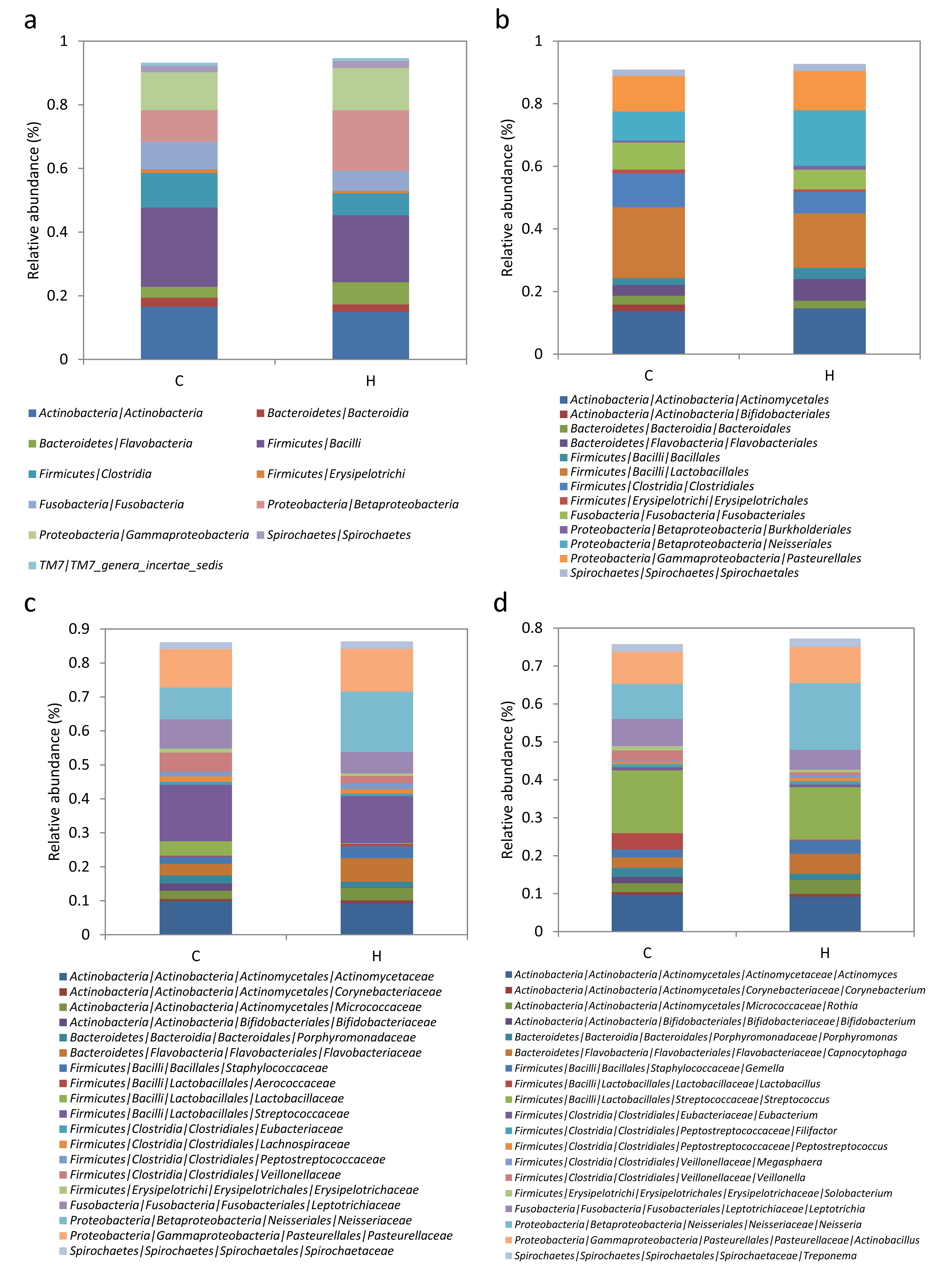

Supplement: S1 Fig — (TIF) [file pone.0147039.s001.tif]

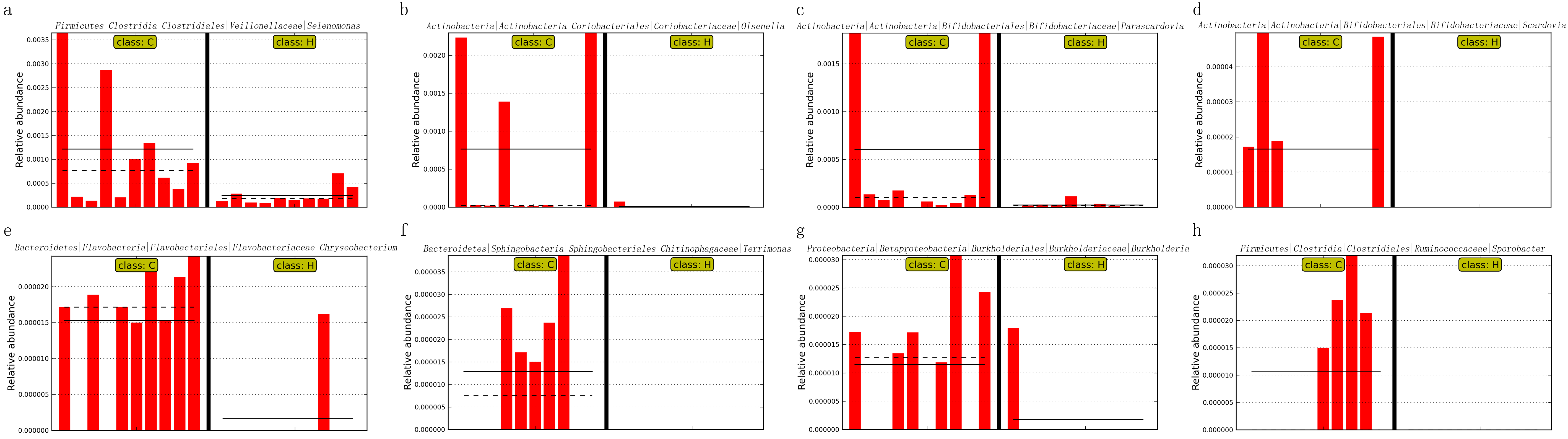

Supplement: S2 Fig — Solid lines and dotted lines represent the means and medians, respectively. (TIF) [file pone.0147039.s002.tif]

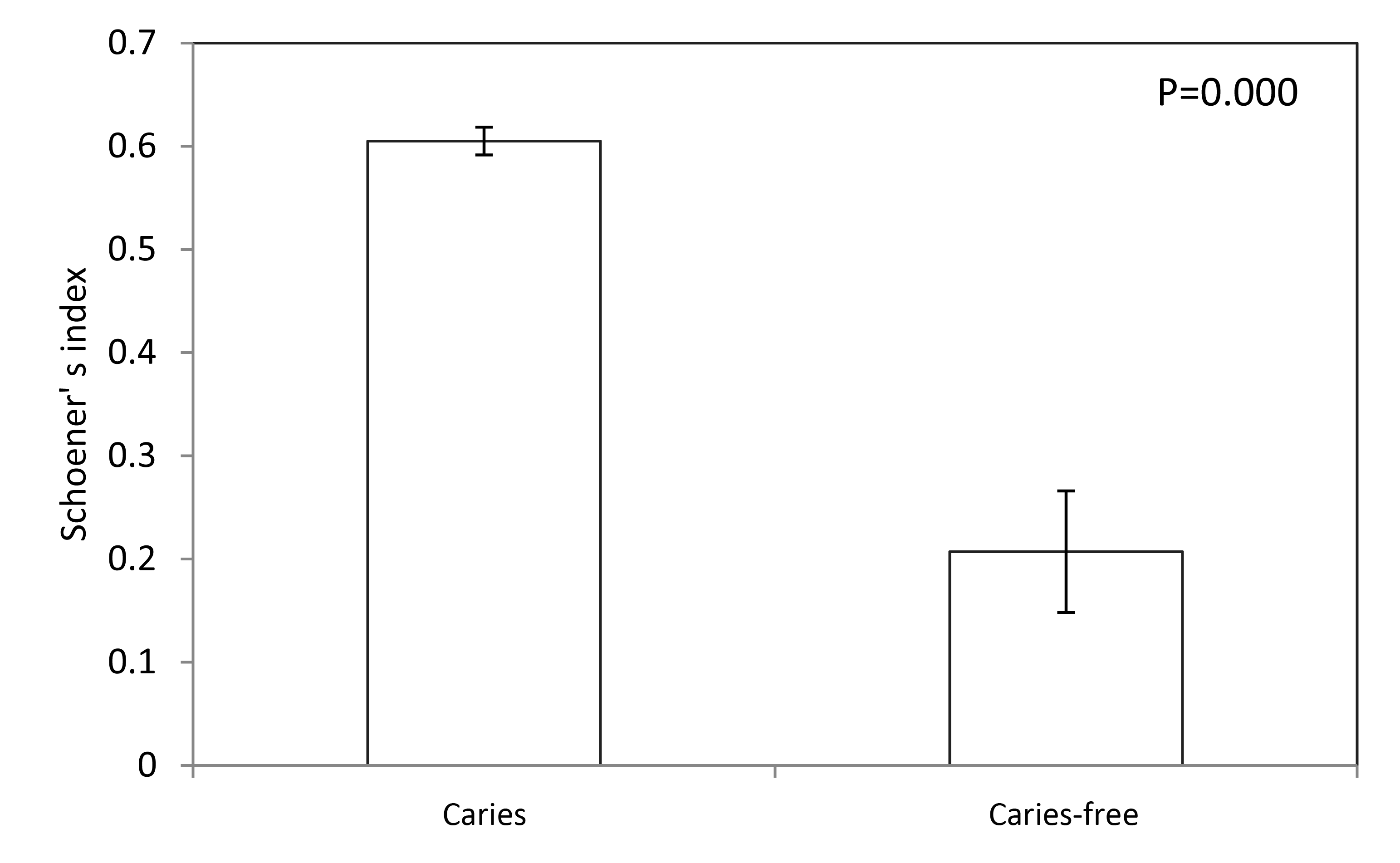

Supplement: S3 Fig — (TIF) [file pone.0147039.s003.tif]
